# Peripheral Cytokine Levels as a Prognostic Indicator in Gastric Cancer: A Review of Existing Literature

**DOI:** 10.3390/biomedicines9121916

**Published:** 2021-12-14

**Authors:** Elton Yang, Wei Chua, Weng Ng, Tara Laurine Roberts

**Affiliations:** 1 School of Medicine, Western Sydney University, Campbelltown 2560, Australia; 19828552@student.westernsydney.edu.au (E.Y.); Wei.Chua@health.nsw.gov.au (W.C.); weng.ng@health.nsw.gov.au (W.N.); 2 Ingham Institute for Applied Medical Research, Liverpool 2170, Australia; 3 Medical Oncology, Liverpool Hospital, Liverpool 2170, Australia; 4 Southwest Sydney Clinical School, University of New South Wales, Liverpool 2170, Australia

**Keywords:** gastric cancer, cytokine, interleukin, tumour necrosis factor, interferon-gamma, *Helicobacter pylori*

## Abstract

Although strong connections exist between the carcinogenesis of gastric cancer and chronic inflammation, gastric cancer is unique in that the chronic gastritis which frequently precedes carcinogenesis is strongly associated with *H. pylori* infection. The interplay between *H. pylori* virulence factors and host immune cells is complex but culminates in the activation of inflammatory pathways and transcription factors such as NF-κB, STAT3, and AP-1, all of which upregulate cytokine production. Due to the key role of cytokines in modulating the immune response against tumour cells as well as possibly stimulating tumour growth and proliferation, different patterns of cytokine secretion may be associated with varying patient outcomes. In relation to gastric cancer, interleukin-6, 8, 10, 17A, TNF, and IFN-γ may have pro-tumour properties, although interleukin-10, TNF, and IFN-γ may have anti-tumour effects. However, due to the lack of studies investigating patient outcomes, only a link between higher interleukin-6 levels and poorer prognosis has been demonstrated. Further investigations which link peripheral cytokine levels to patient prognosis may elucidate important pathological mechanisms in gastric cancer which adversely impact patient survival and allow treatments targeting these processes to be developed.

## 1. Introduction

Gastric cancer (GC) is one of the most common and lethal cancers, ranked as the fifth most common and third most deadly cancer worldwide in 2018 [1]. Current treatment of GC is dependent on tumour, nodes, and metastasis (TNM) staging and involves resection or gastrectomy in resectable cases alongside potential adjuvant chemo/radiotherapy dependant on staging [2]. Recent treatment algorithms have begun to incorporate immunotherapy with some success, including adoptive cell therapy and monoclonal antibodies including immune checkpoint inhibitors [3]. A better understanding of pathways and mechanisms involved in the initiation and progression of GC can elucidate potential therapeutic targets and avenues of treatment, as well as identifying areas where further research is required. Given the vital role of cytokines as signalling and effector molecules in the GC tumour microenvironment (TME) [4,5] and the comparatively limited research in this area, correlations between cytokines and gastric cancer prognosis may allow for better understanding of underlying mechanisms in GC.

The carcinogenesis of gastric cancer marks a unique confluence between extrinsic factors involving the oncogenic potential of cytotoxin-associated antigen A (CagA) positive *H. pylori* [6], and the role of host immune cells and inflammation causing chronic gastritis which may foster an environment favourable to tumourigenesis [7]. Cytokines play a key role within this complex system and may determine the extent of both pro- and anti-tumour interactions and responses [8]. Cytokine production is vital in determining the pathway of T-helper cell differentiation, activation, and regulation of other immune cells [9] and can play a role in tumour growth and metastasis by regulating transcription factors and other intracellular signalling pathways [10]. Consequently, different patterns of cytokine expression may be associated with specific pathways and characteristics of GC progression [11], which may then influence GC patient prognosis and survival.

The production of cytokines that are detectable peripherally during the initiation, and progression of GC may be dependent on *H. pylori* infection and chronic gastritis associated with said infection, as well as variances in the host immune response, particularly that of CD4^+^ T-cells [12]. A discussion on the role of *H. pylori* is presented, followed by a brief review on the effects of CD4^+^ T-cell differentiation in the GC tumour microenvironment (TME) on cytokine levels. A summary of the current findings of the links between individual cytokine levels and the behaviour of gastric tumours concludes this review.

## 2. Pathophysiology of Gastric Cancer

GC is classified in several different ways, although the Lauren classification first proposed in 1965 and the more recent World Health Organisation (WHO) classification in 2010 are the most used [13]. Lauren subdivided GC into intestinal, diffuse, and indeterminate subtypes. The intestinal type is associated with progressive inflammatory change brought about by *H. pylori*, with the subsequent induction of chronic gastritis leading to metaplasia, dysplasia, and finally cancer [7]. Compared to the intestinal subtype, diffuse GC is found in younger patients (more frequently in females), is less prevalent, is linked to mutation of the E-cadherin gene, has no known precursor lesions, and is associated with worse prognosis and tumour severity [14,15,16]. The WHO classification is comprehensive, dividing the Lauren intestinal subtype into papillary, tubular, and mucinous adenocarcinomas, whilst referring to diffuse subtypes as signet cell carcinomas [17]. The WHO classification further includes over 15 additional rare tumour types which can be likened to the Lauren indeterminate classification.

GC treatment is complex, involving surgical resection in most cases as well as adjuvant and neoadjuvant chemo/radiotherapy and is highly dependent on TNM staging [2]. Early gastric cancer is curable with surgical resection, with endoscopic resection used when lymph node involvement risk is low and gastrectomy with lymph node resection in other cases [18]. T1N0 cases do not require adjuvant therapy. In more advanced cases with T2N0 or higher staging, neoadjuvant and adjuvant therapy is required to improve patient outcomes [19]. The chemotherapy backbone typically involves a cytotoxic regime with platinum- and fluoropyrimidine-containing agents [20].

In recent years, additional targeted and immunotherapy approaches have been introduced with treatment dependent on the biomarkers human epidermal growth factor receptor 2 (HER2), programmed death-ligand 1 (PD-L1) as determined by combined positive score (CPS), mismatch repair deficiency (dMMR), and high microsatellite instability (MSI-H) for advanced incurable gastric cancer. Notably, CPS instead of tumour proportion score (TPS) is used; CPS is calculated by counting the total PD-L1 positive tumour cells, lymphocytes, and macrophages, dividing by total tumour cells, then multiplying by 100, whereas TPS omits counting PD-L1 positive lymphocytes and macrophages in its calculation. For HER2 positive tumours, trastuzumab should be added to chemotherapy [21], whilst pembrolizumab should be included in regimes targeting dMMR/MSI-H tumours [22,23]. In HER2-negative patients with PD-L1 CPS ≥ 5, combination chemotherapy with nivolumab is recommended [24].

## 3. Implications of *H. Pylori*

*Helicobacter pylori* is a Gram-negative, spiral bacterium now recognised as one of the most significant risk factors in developing gastric adenocarcinoma [25]. Some strains of *H. pylori* can produce a virulence factor known as cytotoxin-associated gene A (CagA), a 120–145 kDa protein encoded as one of ~30 genes present in a 40 kb DNA segment known as the *cag* pathogenicity island (*cag* PAI) [6]. Approximately 60% of *H. pylori* in the western world are CagA positive; however nearly all *H. pylori* in Southeast and East Asia are CagA positive, a trend that has been linked to the increased prevalence of GC in those geographical regions [26].

**Figure 1:**
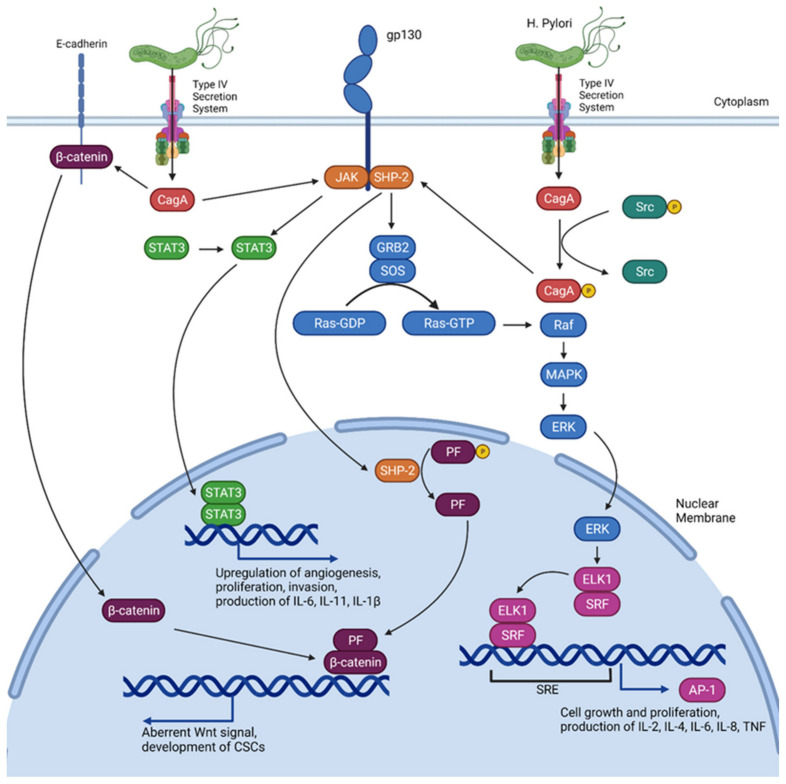
Activation of several inflammatory pathways known to produce cytokines mediated by *H. pylori* infection. CagA injected via T4SS which is phosphorylated by Src activates Ras/Raf/MAPK/ERK via SHP-2 [27]; nuclear translocation of ERK subsequently induces ETS like-1 protein (ELK1) and serum response factor (SRF) binding to the serum response element (SRE) leading to transcription of AP-1 [28]. STAT3 dimerisation and nuclear translocation also occurs after CagA mediated activation of JAK. CagA further activates β-catenin, which in conjunction with SHP-2 dephosphorylating parafibromin (PF) following nuclear translocation enables the formation of the PF/β-catenin complex to activate Wnt signalling pathways [6], which is associated with development of CSCs. AP-1 and STAT3 also play key roles in signalling for cell growth and proliferation; their dysregulation may increase the risk of carcinogenesis.

Upon entering the cytoplasm of gastric epithelial cells via a “molecular syringe” typical of bacterial type IV secretion systems (T4SS), CagA undergoes tyrosine phosphorylation triggering it to act as a mitogen by activating the Ras/Raf/MAPK/ERK pathway [27,28] (a well-known major pro-oncogenic pathway which signals for cellular proliferation). Activation of the MAPK/ERK pathway further induces the “hummingbird” phenotype, which is characterised by an elongated shape reminiscent of epithelial mesenchymal transition (EMT) and increased cell motility [29]. This combination of proliferation and motility gained by gastric epithelial cells is similar to that of cancer stem cells (CSCs) and may be involved in GC carcinogenesis [30]. Finally, MAPK/ERK activation through SH2 containing protein tyrosine phosphatase-2 (SHP-2) can also upregulate cytokine transcription and inflammation within gastric epithelium by upregulating transcription factor activator protein 1 (AP-1), which binds to the promoter region of the tumour necrosis factor (TNF), interleukin-6 (IL-6), and IL-8 genes, amongst others, to further upregulate cytokine production [31,32]. The interaction of *H. pylori* with key inflammatory and pro-oncogenic pathways is summarised in Figure 1.

Another pathway of note is the gp130/JAK/STAT3 pathway which is known to be dysregulated by CagA in a phosphorylation-independent manner [29] (Figure 1). Unphosphorylated CagA is known to activate Janus kinase (JAK) via the gp130 receptor subunit [27], which then phosphorylates signal transducer and activator of transcription 3 (STAT3). Subsequently, STAT3 undergoes dimerisation and translocates to the cell nucleus where it downregulates genes controlling apoptosis and upregulates genes involved in cell proliferation and inflammation, including the production of IL-6, IL-1β, and IL-11 [33]. Notably, this pathway is usually activated via IL-6 binding to the IL-6 receptor and subsequent complexation with glycoprotein 130 (gp130). Therefore, CagA dysregulation of this pathway may exaggerate its activation and augment the pro-oncogenic effects of IL-6, as discussed later [34,35]. Finally, STAT3 is also involved in T helper cell 17 (Th17) cell differentiation, which is also discussed later in the review [36].

**Figure 2:**
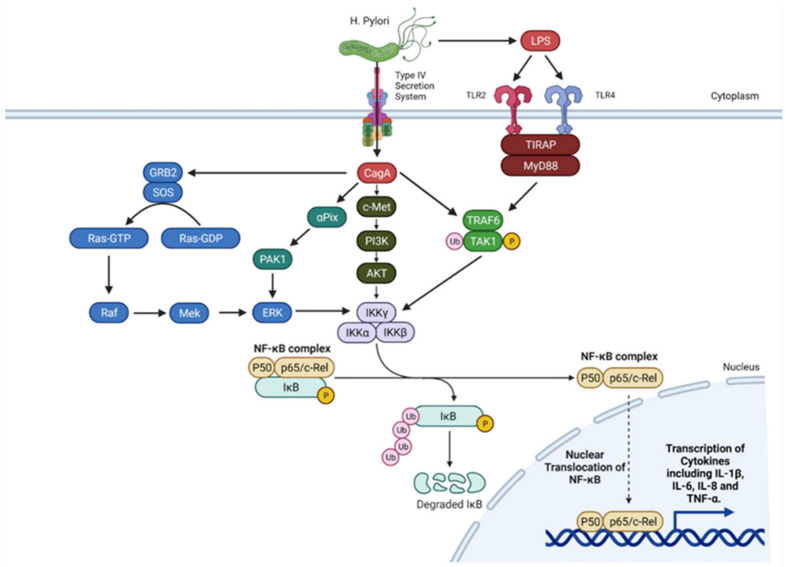
Activation of NF-κB by CagA released by *H. pylori.* Multiple activation pathways are CagA dependent; activation of growth factor receptor-bound protein 2 (GRB2) leads to Ras/Raf activation, which in turn activates NF-κB. This pathway can also occur via P21-activated kinase 1 (PAK1) [32]. NF-κB activation can also occur through c-met and phosphatidylinositol 3-kinase (PI3K) upstream activation, as well as via tumour necrosis factor receptor-associated factor 6 (TRAF6) and transforming growth factor-β-activated kinase 1 (TAK1). The TRAF6/TAK1 pathway can be activated by *H. pylori* liposaccharide (LPS) activation of toll-like receptor 4 (TLR4). Figure adapted from “NF-κB Signalling Pathway”, by BioRender.com (2021). Retrieved from https://app.biorender.com/biorender-templates (accessed on 6 November 2021).

*H. pylori* also activates nuclear factor kappa B (NF-κB), a transcription factor which plays a key role in signalling inflammation [32,37,38] (Figure 2). Typically, NF-κB is kept inactivated in the cytoplasm by being bound to IkB (inhibitor of NF-κB). Upon upstream pathway activation, IkB is degraded by IKK (IkB kinase), allowing NF-κB to translocate to the nucleus and upregulate gene transcription through binding to its target sequence in the promoter of target genes. There have been numerous proposed mechanisms for the upregulation of NF-κB activity by *H. pylori* which include pathways both dependent and independent of CagA, T4SS, peptidoglycans, and other virulence factors of *H. pylori* such as urea. Although the exact mechanism is unclear, many of these mechanisms likely contribute to production of cytokines regulated by NF-κB including IL-1β, IL-6, TNF-α [39], and IL-8 [40].

Overall, these combined pathways contribute to the ability of *H. pylori* to induce a state of chronic inflammation through activating inflammatory mediators, with CagA being a key molecule in this process [41]. This leads to the upregulation of various oncogenic pathways and the production of various cytokines, the effects of which are discussed later.

## 4. Effects of Immune Cell Differentiation in Gastric Cancer

Immune cells within the tumour microenvironment (TME) substantially affect tumour behaviour and impact on disease progression. CD4^+^ T-cells, also known as T-helper cells, modulate immune responses to GC through their key role in signalling other immune cells via cytokine release [12]. Furthermore, gastritis and inflammation characteristic of chronic *H. pylori* infection leads to oncogenic change which is largely mediated through CD4^+^ T-cells [10], highlighting their importance in carcinogenesis as well as cancer progression.

**Figure 3:**
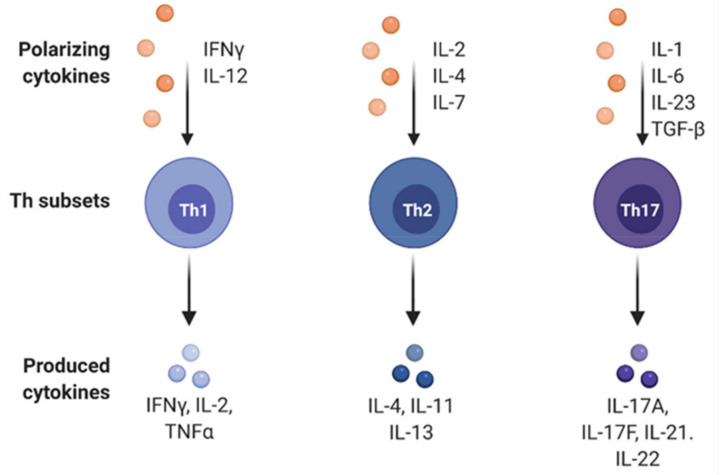
Differentiation pathways of CD4^+^ T-cells, which are dependent on the cytokine profile. Consequently, each different CD4^+^ T-cell lineage possesses a distinctive cytokine production pattern as shown above. Figure adapted from “T cell activation and differentiation”, by BioRender.com (2021). Retrieved from https://app.biorender.com/biorender-template (accessed on 6 November 2021).

The hypothesis that CD4^+^ T-cells differentiate into distinct patterns known as Th1/Th2 cells arose from observations that subpopulations of CD4^+^ T-cells had differing patterns of cytokine release (Figure 3). Cytokines are essential in determining which lineage naïve T-cells mature into; Th1 cells require IL-12 and interferon gamma (IFN-γ), whereas Th2 cells require IL-4 and IL-2/IL-7. Subsequently, Th1/Th2 cells secrete cytokines dependent on their lineage. Th1 cells characteristically secrete IFN-γ and IL-2 but also TNF-α. In contrast, Th2 cells secrete IL-4, IL-11, and IL-13 [42,43]. Therefore, the predominance of the T-helper cell type influences the composition of cytokine secretion and the behaviour of immune cells. In *H. pylori* infection, reports have suggested a Th1 predominant response. This is substantiated by Meyer et al. [44] who demonstrated significant IFN-γ production but a lack of IL-2 expression when peripheral blood mononuclear cells (PBMCs) were cultured with *H. pylori*; such a cytokine profile would induce Th1 differentiation but inhibit differentiation into the Th2 subtype.

Studies including Ubukata et al. [45] revealed a correlation between Th1/Th2 predominance in peripheral blood samples and prognosis in a study involving 157 patients, showing that Th1/Th2 ratio ≥ 8 (indicating Th1 predominance) was linked to 5-year survival of 78.4% as opposed to 21.2% in patients with Th1/Th2 ratios < 8. Ubukata et al. further showed that patients whose Th1/Th2 ratio decreased throughout disease progression had worsening prognosis compared to patients who maintained a high ratio throughout (16% vs. 94% 5-year survival rates), exposing a possible shift towards Th2 predominance in cancer progression. However, the mechanism for this shift, as well as the link between T-helper cell predominance and cancer prognosis, remains unclear but may be linked to changing compositions of cytokine secretion.

A more recently discovered lineage of CD4^+^ cells known as Th17 cells has been studied for possible correlation with GC progression. Naïve T-cells differentiate into Th17 cells following exposure to TGF-β and IL-6 and possibly IL-1 and IL-23, and Th17 cells characteristically secrete IL-17A, IL-17F, IL-21, and IL-22 [36].

Although studies have shown a relationship between Th17 cells levels and tumour progression, thus implying a pro-tumour role of Th17 cells [46,47], these results have not been without controversy. Notably, one study showed that increased IL-17^+^ cells (encompassing both Th17 cells and CD8^+^ IL-17^+^ cells) within the GC TME improved prognosis [48]. In contrast, other studies have shown the opposite, that increased Th17 cells in serum adversely impact OS or TNM staging [49,50].

Literature surrounding CD4^+^ T-cells in the TME remains controversial, which is compounded by the lack of literature with a specific focus on the role of CD4^+^ T-cells within GC. Although a high Th1/Th2 ratio in peripheral blood may be associated with an improved prognosis, the exact mechanisms regulating this balance remain unknown, although differing patterns of cytokine secretion depending on Th1/Th2 predominance may contribute to said mechanism. Finally, there remains mixed evidence around the effects of Th17 on prognosis, and further research is needed to clarify this discrepancy.

## 5. Effects of Cytokines on Gastric Cancer Prognosis

This section will focus on key cytokines involved in immune response polarisation and their potential tole in GC biology and prognosis. Relevant studies for the key cytokines addressed here are summarised in Table 1.

### 5.1. IL-6

Interleukin-6 (IL-6) has numerous pro-inflammatory roles and regulates multiple pathways in cancer associated with poor prognosis [51]. Within the GC TME, IL-6 is produced by tumour-associated fibroblasts [52,53] and tumour cells as well as immune cells [54]. One of the most important activators of the gp130-JAK-STAT3 pathway is IL-6 [27,55], with STAT3 known to induce tumour growth and immunosuppression. STAT-3 promotes the transcription of factors involved in inflammation (IL-6, IL-11, and IL-1β) [33], angiogenesis (VEGF), invasion (MMPs), and regulators of cell proliferation (cyclin D1, MYC) [56], all of which mediate pro-tumour effects. As mentioned, STAT3 is greatly upregulated by *H. pylori* infection [34]; this may exacerbate its oncogenic potential specifically in GC.

Interestingly, IL-6 is exploited by GC tumour cells to aid in their proliferation. Zhu et al. [57] demonstrated the existence of cross-talk between GC mesenchymal stem cells (MSCs) and tumour-associated neutrophils mediated by IL-6 and STAT3. When GC MSCs secrete IL-6 to activate neutrophils and inhibit their apoptosis, the GC-associated neutrophils in turn induce differentiation of GC MSCs into cancer-associated fibroblasts (CAFs) which may produce even more IL-6 [53]. CAFs are an important component of the TME and play a key role in GC progression, possibly through enhancing tumour migration, angiogenesis, metastasis, and chemotherapy resistance, though the mechanisms remain unclear [53,58]. Another pathway involves the production of hepatocyte growth factor (HGF) by interstitial cells within the TME when exposed to IL-6 [59,60]. HGF may then activate c-met (HGF receptor) on tumour cells which then increases mitogenic and motogenic activity.

IL-6 receptor (IL-6R) expression may also be significantly increased in gastric tumour tissue compared to normal tissue [59]. IL-6R expression is especially increased in advanced gastric cancer, and elevated levels are associated with a poor prognosis. Multiple studies have examined levels of IL-6 in GC patient serum versus healthy controls; all demonstrated increased IL-6 in GC patients [61,62,63]. A systematic review by Vainer et al. [64] yielded 10 papers which investigated IL-6 in GC; six papers assessed the severity of GC clinical characteristics (including tumour size, invasion, and metastasis) with IL-6 levels, and all six found a correlation between GC severity and increased IL-6. Similar trends were seen between IL-6 and OS, with one study by Ashizawa et al. [60] demonstrating GC survival rates of 43% and 87% in patients with high and low serum levels of IL-6, respectively (*n* = 60). Another study by Liao et al. [65] showed that in patients with stage II or III GC (*n* = 86), survival time was significantly longer (1418 days) for patients with serum IL-6 levels ≤13 pg/mL, as opposed to 618 days in patients with serum IL-6 levels >13 pg/mL.

Evidence links increased IL-6 levels in both tissue and serum with worsened clinical characteristics and prognosis. Although there are numerous proposed mechanisms justifying this link, further research could better elucidate these pathways, with focus on how they could be therapeutically targeted.

### 5.2. TNF

Tumour necrosis factor (TNF) is a pro-inflammatory cytokine, so named for its capability to induce tumour haemorrhagic necrosis [66]. The origins of TNF in the GC TME are unclear, but epithelial and stromal cells are thought to be a source in addition to infiltrating immune cells [67]. As a key inflammatory mediator, TNF is able to induce tumour cell death and inhibit tumour proliferation [68]. However, TNF exerts many of its actions through TNF receptor 1 (TNFR1) which upregulates downstream pro-inflammatory pathways such as NF-κB, AP-1, IL-8, vascular endothelial growth factor (VEGF), and matrix metalloproteinases (MMPs) implicated in tumour survival, angiogenesis, and migration [67,69]. Consequently, the role of TNF in carcinogenesis has garnered attention in recent years.

One example of a TNF-mediated pro-oncogenic mechanism involves the action of TNFR1 to upregulate Noxo14 [70], which encodes NOX-organising protein 1, a component of NADPH oxidase 1 (NOX1). NOX1 is an enzyme known to generate reactive oxidative species (ROS) which can be favourable for oncogenesis by activating inflammatory mediators within the gastric mucosa [71], causing tissue damage and subsequent increased DNA damage.

In GC, TNF may be especially relevant as a pro-inflammatory factor. The TNF-α-inducing protein (tipα) within *H. pylori* is a separate virulence factor to CagA; tipα complexes with cell surface nucleolin which is then internalised, leading to strong expression of TNF-α [72]. Furthermore, TNF-α polymorphisms, especially TNF-α-308 G/A, are linked to a substantial increase in risk of developing gastric cancer [73,74], although the mechanism is unknown. TNF thus has the potential to act as a double-edged sword with both pro- and anti-tumour action dependent on the surrounding environment and balance between activation and feedback inhibitory signals.

Despite TNF’s anti-tumour actions, TNF is involved in a range of inflammatory and pro-tumour pathways which may lead to poor prognosis in GC. This may be exacerbated by specific polymorphisms with increased oncogenic activity and increased uptake of TNF through novel *H. pylori* virulence factors.

### 5.3. IFN-γ

Interferon-gamma (IFN-γ) is a key cytokine involved in cellular immunity and associated with both pro- and anti-tumour activity. It is involved in numerous disease-modifying processes, such as recruitment and development of immune cells, recognition of tumour cells by immune cells, and regulation of apoptosis [75,76]. IFN-γ is produced by Th1 cells as well as CD8^+^ cytotoxic T-cells and NK cells and acts to cause Th1 differentiation whilst simultaneously suppressing Th2 differentiation [77]. IFN-γ activates the JAK/STAT1 pathway, which in turn activates IRF-1 to stimulate MHC class I antigen presentation [78]. MHC class I is vital for immune system recognition and elimination of malignant cells. Despite its effect in inducing Th1 differentiation, however, Tu et al. demonstrated that a 2- to 3-fold increase in IFN-γ expression in murine gastric mucosa could instead induce apoptosis in CD4^+^ T-cells, reducing immune responses within Th1 and Th17 cells [79], which has a protective effect against tumourigenesis by reducing pro-tumour cytokine production and inflammation.

However, there exists evidence of IFN-γ acting as a pro-oncogenic factor. The same JAK/STAT1 pathway activated by IFN-γ also upregulates programmed death-ligand 1 (PD-L1) expression on gastric tumour cells [80]. PD-L1 is a checkpoint protein which binds to PD-1 on activated T- and B-cells, especially CD8^+^ cells, to suppress their anti-tumour response [81], thereby ensuring tumour cell survival and evasion. Therefore, chronic activation of IFN-γ can in fact lead to a predominant activation of negative feedback pathways which suppress anti-tumour immunity.

Furthermore, IFN-γ has been reported to enhance inflammatory responses in gastric epithelial cells to CagA+ *H. pylori* mediated by nucleotide oligomerization domain 1 (NOD1) [82]. NOD1 acts to recognise bacterial molecules such as peptidoglycan within cells and can be chronically active in CagA+ *H. pylori* infection often seen in GC [83]; NOD1 then activates potent inflammatory mediators such as NF-κB. IFN-γ augments this process by increasing NOD1 expression, which may have pro-tumour consequences, exacerbated by the presence of CagA.

Overall, there is mixed evidence pointing towards mechanisms which drive both pro- and anti-tumour activity of IFN-γ. However, there were no studies which were found to investigate the relationship between patient prognosis and IFN-γ levels. There is weak evidence, however, of higher serum levels of IFN-γ leading to higher incidence rate of GC as documented by Sánchez-Zauco et al. [61].

### 5.4. IL-17A

Interleukin-17A (IL-17A), part of the IL-17 family [84], is a pro-inflammatory cytokine increasingly recognised in tumour initiation and growth [85]. It is characteristically produced by Th17 cells but also by some neutrophils, NK, CD8^+^ T-cells, and CAFs [86,87,88] within the gastric cancer TME.

NF-κB is reported as one of the downstream targets of IL-17A in other cell types [89]. Aside from the pro-oncogenic effects of NF-κB previously discussed, IL-17A in conjunction with NF-κB specifically increases matrix metallopeptidase 2 (MMP-2) and MMP-9 expression in GC [90]. MMPs degrade the extracellular matrix (ECM) which is an essential step in tumour invasion and metastasis [91].

Further, IL-17A upregulates the production of VEGF via a STAT3 dependent pathway, likely the IL-17a/JAK2/STAT3 pathway [92]. Consequently, studies have demonstrated a direct relationship between IL-17 levels in serum and tumour microvessel density (MVD) [86], associated with high levels of angiogenesis and potentially associated with tumour growth and poor prognosis [93].

Activation of STAT3 by IL-17A impacts quiescent gastric cancer stem cells (CSCs), which showed EMT-like transformation after exposure to IL-17A [94]. Specifically, decreased expression of E-cadherin and increased expression of vimentin and N-cadherin were observed as well as increased invasion and migration capabilities, which is characteristic of EMT. Additionally, increased levels of tumour-associated neutrophils (TANs) have been linked to GC, and TANs which produce IL-17A have been associated with EMT of GC cells and predict poor prognosis [95]. Furthermore, IL-17A may be involved in recruitment of neutrophils, which then induce angiogenesis [96], possibly through the JAK2/STAT3 pathway discussed above. IL-17A production has also been demonstrated by CAFs, with one study by Zhang et al. [87] showing that concordant increased levels of CAFs and IL-17A were associated with advanced TNM staging and poorer patient outcomes.

IL-17A exerts pro-oncogenic effects through multiple pathways. However, few studies have investigated the relationship between serum IL-17 levels and GC survival, and the studies available remain controversial. Notably, a meta-analysis which investigated the relationship between IL-17 and tumour progression and overall survival by Zeng et al. [97] concluded that there was no statistically significant relationship between IL-17 and worse prognosis in GC. Chen et al. [98] further concluded that high levels of intratumoural IL-17 were associated with improved prognosis (*n* = 192).

Conversely, other studies have concluded that increased levels of IL-17 and Th17 cells in serum adversely impacts OS [47]. Moreover, Iida et al. [99] (*n* = 82) found that increased levels of IL-17 mRNA in GC tissue were associated with increased tumour depth and lymph node involvement. In order to resolve these conflicting results, more studies on this topic should be undertaken to investigate if a potential correlation exists and determine if the role of IL-17 differs between subtypes of GC or is related to the presence of *H. pylori* infection.

### 5.5. IL-10

Interleukin-10 (IL-10) is one of the most important anti-inflammatory cytokines playing a key role in immunoregulation [100]. In GC, IL-10 is secreted by both tumour-associated macrophages [101] (TAMs) and T_reg_ cells [102], although many immune effector cells produce IL-10 to some extent [103]. The role of IL-10 in cancer has proven highly controversial with various contradictory findings.

IL-10 was traditionally thought to promote tumour proliferation by inhibiting immune responses [104], with studies revealing positive correlation between IL-10 levels and tumour proliferation in various malignancies [105,106]. IL-10 exerts regulatory effects on inflammatory pathways involving STAT3 and NF-κB, which may influence tumour progression. However, other studies have shown tumour regression in murine models following IL-10 secretion. One hypothesis is that IL-10 stimulates NK activity under certain conditions [107], which allows for cancer cell destruction and tumour regression; however, this tumour regression may not be replicable in humans due to species-specific differences in IL-10 or other related immune pathways.

Unfortunately, few recent studies have investigated the effects of IL-10 expression specifically in relation to gastric cancer. Tang et al. [108] explored methylation of CpG islands within the IL-10 gene in GC tumour and adjacent tissue. Hypermethylation of CpG is associated with decreased gene expression; hypomethylation results in the reverse effect. Tang demonstrated that hypomethylation of CpG islands within the IL-10 gene in GC tumours and adjacent tissue was associated with decreased OS, thus linking increased IL-10 expression with poor prognosis.

Further, Chen et al. [101] scrutinised the oncogenic role of TAMs in GC finding that IL-10 was expressed in the cell culture supernatant of GC TAMs, and that exposing tumour cells to said supernatant increased tumour proliferation. Introduction of anti-IL10 antibody partially blocked proliferation induced by the supernatant, supporting a causative link between IL-10 and tumour proliferation.

Although studies have demonstrated a link between worsened outcome and IL-10 expression, the lack of studies on human GC tissue means this link remains inconclusive.

### 5.6. IL-8

Interleukin-8 (IL-8), also known as CXCL8, is a member of the CXC family of chemokines [109]. IL-8 is best known for its chemoattractant properties for neutrophils; more relevant to GC, however, are its pro-inflammatory properties and a strong association with angiogenesis and tumour metastasis [110]. In the GC TME, IL-8 is overexpressed; one explanation is that production is upregulated by CagA+ *H. pylori* in gastric epithelial cells [111], possibly via the NF-κB and AP-1 pathways [40,112] as discussed above. IL-8 is additionally produced by macrophages and neutrophils [113], which are often present in the GC TME. Tumour-derived IL-8 has also been documented in studies via an autocrine pathway [109,114]. IL-8 activation of CXCR1/CXCR2 (Interleukin-8 receptor) activates the Ras/Raf/Mek/Erk pathway which, in turn, upregulates IL-8 transcription.

Given IL-8’s association with angiogenesis, studies such as that of Kitadai et al. have shown a positive correlation between IL-8 expression and tumour vascularisation in humans [115]. Unfortunately, the exact mechanism for angiogenesis remains controversial; despite studies reporting that IL-8 induces VEGF expression via CXCR2 in a murine model [116], Kido et al. presents a contrary view, instead reporting no correlation between IL-8 and VEGF levels in GC tumour sections (*n* = 56) and elevated levels of IL-8 but normal VEGF amongst human GC mucosa compared to control [117]. Another study by Yeni et al. (*n* = 45) reported positive correlation between IL-8 and VEGF levels, but under-expression of IL-8 and VEGF in human GC mucosa compared to control [118]. Despite these conflicting results, it remains likely that IL-8 induces angiogenesis in GC given the undisputed reports of increased tumour vascularisation [115,116,119]. However, the exact mechanism remains controversial for the moment and may not involve VEGF as previously thought.

IL-8 has also been implicated in EMT. Ju et al. showed that IL-8 upregulated MMP-9 and intercellular adhesion molecule 1 (ICAM-1) expression in humans as well as downregulating E-cadherin levels, allowing for tumour cell migration and invasion [120]. Tumour-derived IL-8 can promote metastasis when receptor for activated C-kinase 1 (RACK1) is dysregulated [121], highlighting IL-8’s potential for worsened prognosis. Furthermore, IL-8 also upregulates immune evasion, with IL-8 inducing PD-L1 overexpression within macrophages and decreasing CD8^+^ cytotoxic T-cell infiltration in the human GC TME [122], which may enhance tumour cell survival and worsen patient outcomes.

Finally, CXCR1 overexpression has been linked to GC progression, with Hu et al. demonstrating increased levels of CXCR1 in late-stage gastric adenocarcinoma and that knockdown of CXCR1 in GC cells may inhibit tumour proliferation [123]. This is especially significant given CXCR1’s high specificity in binding IL-8 with nanomolar affinity [124].

It is apparent that IL-8 has been implicated in a number of pro-oncogenic and pro-tumour pathways, upregulating tumour cell migration, invasion, metastasis, survival, immune evasion, and angiogenesis. However, few studies have investigated the effect of IL-8 expression on prognosis specifically in GC. Kido et al. did demonstrate decreased OS in GC patients with high levels of IL-8 compared to low IL-8 (*n* = 56), but statistical significance was not reached [117]. Further studies investigating a potential link between IL-8 expression and survival rates are needed to address this gap in literature.

## 6. Discussion

The impact of cytokines on tumour progression and prognosis in GC is complex and multifactorial. Cytokine production within the GC TME arises from multiple sources, including downstream targets of *H. pylori* virulence factor CagA, host immune cells and especially CD4^+^ T-cells, and tumour cells. Pathways regulating cytokine secretion are likewise complex, although the NF-κB, STAT3 and AP-1 transcription factors stand as pillars in this network. However, a multitude of smaller pathways, including novel ones such as reprogramming and crosstalk between TAMs and TANs, and tumour cells complicate the overall picture. CD4^+^ T-cells, however, represent a significant portion of cytokine production. Th1/Th2/Th17 balance, therefore, alters cytokine expression patterns within the GC TME and impacts prognosis, with higher Th2 to Th1 ratios associated with poor outcomes. However, since CD4^+^ T-cells secrete multiple cytokines and display plasticity and heterogeneity, it is difficult to decouple and isolate the impacts of specific cytokines. Studies generally also only investigated CD4^+^ T-cells belonging to either the TME or the periphery and not both, making connections between peripheral cytokine levels and tumour-associated immune cells difficult to establish. Discrepancies in correlation between tumour and circulating cytokine levels may underly some contradictory data in the literature. However, these types of studies are particularly difficult in humans as repeated biopsies on patients across the disease progression is not part of standard clinical care. For this reason, analysis of peripheral cytokines is required to examine role at different stages of the disease.

There was evidence that all cytokines investigated, in relation to GC, have pro-tumour effects, although TNF, IFN-γ, and IL-10 may also participate in anti-tumour processes. However, the lack of studies which specifically investigated the role of cytokines as prognostic factors in GC and the low statistical power of some studies hinders any definitive conclusions. IL-6 was the only cytokine shown to correlate with poorer outcomes; six papers assessed within the systematic review by Vainer et al. [64] linked decreased OS to increased IL-6. Confounders within the included papers include small sample sizes, with one investigating only 51, as well as large variances of IL-6 cut-off ranges between “low” and “high” groups, ranging from 0.8 to 50 pg/mL; these factors introduce a factor of uncertainty to the conclusion. Disappointingly, no studies which investigated the prognostic role of TNF, IFN-γ, or IL-10 were found, although some studies did show the value of IFN-γ and IL-10 as a diagnostic marker for GC [61]. Therefore, a gap in the literature exists for the prognostic role of these cytokines within GC which future studies may address.

Notably, differing IL-17A and IL-8 levels were shown by some studies to not affect patient prognosis, despite evidence suggesting multiple pro-tumour pathways upregulated by these cytokines in GC. An explanation for these results is that the pathways upregulated by IL-17A and IL-8 are already activated by other factors within the TME, and so the presence of IL-17A and IL-8 does not significantly affect the extent of activation of these pathways. However, this explanation is contraindicated by multiple studies investigating the mechanism of pro-tumour effects of cytokines [95,121]. These studies demonstrated significant decreases in tumour aggression when antibodies against cytokines/cytokine receptors were introduced, suggesting that cytokines alone may upregulate pro-tumour signals. These studies, however, were largely performed in vitro and so may lack many mechanisms and factors present within human GC TME, confounding results. Further, many studies used an arbitrary cut-off point to define “high” vs. “low” cytokine levels used to drive statistical analysis. However, this does not account for the changes in individual immune profiles over age; the lack of age-adjustment in cut-offs means that these results may not be generalisable for younger individuals who may have a different baseline immune response and therefore differing cytokine levels.

Furthermore, statistical deficiencies further confounded findings for IL-8 and IL-17A. For example, Kido et al. [117] demonstrated that higher IL-8 levels were linked to lower OS, but this result was statistically insignificant (*p* = 0.08). However, the study power was calculated to be 0.67, below the generally accepted threshold of 0.8. The meta-analysis performed by Zeng et al. [97] identified four studies involving IL-17 and gastric tumours and found no statistically significant connection between IL-17 levels and OS; this may be due to small sample size or higher variability between studies, and more studies are required to confirm this conclusion. Furthermore, three out of four included studies did not measure IL-17 levels directly but instead measured Th17 cell count, which could be a potential confounding factor.

Despite cytokines being involved in complex mechanisms which contribute to GC progression, compounded by the inflammatory state induced by *H. pylori* seen in many GC patients, there have been few studies which measured the impacts of cytokines on patient prognosis. Higher levels of IL-6 were shown to have a degree of correlation with poorer outcomes, but the lack of high-quality evidence for other cytokines means any links to prognosis remain inconclusive. Further studies may allow for relationships between cytokine levels and prognosis to be better understood, which may uncover the importance of certain cytokines within GC progression and/or the response to specific therapies. Further, the impact of current therapeutic regimes on cytokines profiles needs to be assessed particularly as immune modulating treatment such as immune checkpoint inhibitors are introduced into care of patients with GC. Pathways involving these cytokines can then be more closely examined which may elucidate new ideas on the progression of GC, the relative importance of specific mechanisms, and innovative treatment targets.

## Figures and Tables

**Table 1:** Summary of cytokine effects and mechanisms affecting GC progression, sources in GC, and effect on prognosis.

Cytokine	Overall Effect	Sources within GC TME	Mechanisms	Effect on Prognosis
IL-6	Pro-tumour	Tumour-associated fibroblasts, tumour cells [51,52,54]	Activates its receptor complexed with gp130, which activates JAK-STAT3 [25,55]. STAT3 is known to promote the transcription of factors involved in inflammation such as IL-6, IL-11, and IL-1β [33], angiogenesis (VEGF), invasion (MMP2), and cell survival (cyclin D1, MYC) [56]. Secreted by GC MSCs to allow for tumour-associated neutrophil (TAN) proliferation [57]; TANs in turn induced differentiation of GC-MSCs to cancer-associated fibroblasts (CAFs) which are implicated in cancer progression [53,58]. Induces production of HGF by interstitial cells within the GC TME to activate c-met, which increases mitogenic and motogenic activity [59,60].	IL-6 serum levels and IL-6R levels in GC tissue have been linked to GC severity and poorer OS. A systematic review by Vainer et al. [64] Found that all six papers which assessed clinical characteristics of GC and IL-6 levels linked decreased OS to increased IL-6. Ashizawa et al. [60] Demonstrated GC survival rates of 43% and 87% in patients with high and low serum levels of IL-6, respectively.
OS	Pro- and anti-tumour	Epithelial Cells, stromal cells [67]	Induces tumour haemorrhagic necrosis [66] and tumour cell death and inhibits tumour proliferation [68]. TNF receptor 1 (TNFR1) upregulates pro-inflammatory pathways such as NF-κB, AP-1, IL-8, VEGF, and MMPs implicated in tumour survival, angiogenesis, and migration [69]. Upregulation of Noxo14, which generates ROS and is thus favourable for oncogenesis [70,73,74]. Some TNF-α polymorphisms have been associated with increased GC risk.	No studies which investigated the relationship between TNF levels and GC patient prognosis were found.
IFN-γ	Pro- and anti-tumour	CD4^+^ Th1 and CD8^+^ cytotoxic T-cells, NK cells [77]	Activation of Jak/STAT1/IRF-1 which stimulates MHC class I recognition of malignant cells [78]. Upregulation of PD-L1 expression on GC cells allowing for immune evasion [80]. Activation and enhancing detection of *H. pylori* peptidoglycan by NOD1, which in turn upregulates NF-κB pathways [82,83].	N Sánchez-Zauco. et al. [61] showed that high plasma/serum levels of IFN-γ are associated with GC incidence rate. No studies were found to investigate the relationship between IFN-γ levels and GC patient prognosis.
IL-17A	Pro-tumour	CD4^+^ Th17 and CD8^+^ cytotoxic T-cells, NK cells, neutrophils [86,87,88]	Upregulation of NF-κB, and specifically increase in MMP-2 and MMP-9 expression which aids in tumour metastasis [89,90]. Upregulate VEGF production via STAT3 and increases tumour microvessel density (MVD) which was associated with increased metastasis risk [86,92]. STAT3 activation leads to decreased expression of E-cadherin and increased expression of vimentin and N-cadherin characteristic of EMT, as well as increased invasion and migration capabilities [94]. Increased levels of tumour-associated neutrophils (TANs) which in turn have been associated with poor prognosis [95,96].	Mixed evidence: Iida et al. [99] (*n* = 82) linked increased IL-17 mRNA to increased tumour depth and lymph node involvement, but a meta-analysis by Zeng et al. [97] concluded that there was no statistical significance between IL-17 and worsened prognosis in GC.
IL-10	Pro- and anti-tumour	CD4^+^ Th1 and Th2 cells, Treg cells, TAMs [101,102,103]	Hypomethylation of IL-10 gene was associated with increased GC risk and decreased OS [108]. IL-10 is expressed in the cell culture supernatant of GC TAMs, and the supernatant induced tumour growth and proliferation [101].	No studies which investigated the relationship between levels of IL-10 and GC prognosis/outcome were found.
IL-8	Pro-tumour	Macrophages, neutrophils [113], tumour cells [109,114], epithelial cells [111]	Increase in tumour vascularisation [115,116,119]. Increase in tumour migration and invasion by upregulating MMP-9 and ICAM-1, and downregulating E-cadherin [120]. Upregulation of immune evasion by inducing PD-L1 expression on macrophages [122]. Increased levels of CXCR1 (interleukin-8 receptor) are linked to worsened prognosis [123].	Limited evidence available. Kido et al. showed decreased OS in GC patients with high levels of IL-8 vs. low IL-8 (*n* = 56), but results were not statistically significant [117].

## Data Availability

Not applicable.
